# Early detection of cotton verticillium wilt based on root magnetic resonance images

**DOI:** 10.3389/fpls.2023.1135718

**Published:** 2023-03-20

**Authors:** Wentan Tang, Na Wu, Qinlin Xiao, Sishi Chen, Pan Gao, Yong He, Lei Feng

**Affiliations:** ^1^ College of Biosystems Engineering and Food Science, Zhejiang University, Hangzhou, China; ^2^ Key Laboratory of Spectroscopy Sensing, Ministry of Agriculture and Rural Affairs, Hangzhou, China; ^3^ School of Information and Electronic Engineering, Zhejiang University of Science and Technology, Hangzhou, China; ^4^ College of Information Science and Technology, Shihezi University, Shihezi, China

**Keywords:** cotton, verticillium wilt, root, MRI, detection, SwinUNet, ResNet

## Abstract

Verticillium wilt (VW) is often referred to as the cancer of cotton and it has a detrimental effect on cotton yield and quality. Since the root system is the first to be infested, it is feasible to detect VW by root analysis in the early stages of the disease. In recent years, with the update of computing equipment and the emergence of large-scale high-quality data sets, deep learning has achieved remarkable results in computer vision tasks. However, in some specific areas, such as cotton root MRI image task processing, it will bring some challenges. For example, the data imbalance problem (there is a serious imbalance between the cotton root and the background in the segmentation task) makes it difficult for existing algorithms to segment the target. In this paper, we proposed two new methods to solve these problems. The effectiveness of the algorithms was verified by experimental results. The results showed that the new segmentation model improved the Dice and mIoU by 46% and 44% compared with the original model. And this model could segment MRI images of rapeseed root cross-sections well with good robustness and scalability. The new classification model improved the accuracy by 34.9% over the original model. The recall score and F1 score increased by 59% and 42%, respectively. The results of this paper indicate that MRI and deep learning have the potential for non-destructive early detection of VW diseases in cotton.

## 1 Introduction

Cotton is an essential cash crop. Unfortunately, cotton’s growth can be affected by numerous diseases, with Verticillium wilt (VW) being the most destructive (Billah et al., 2021). VW is a systemic disease of the entire reproductive period, with symptoms typically appearing after bud emergence and peaking during flowering and boll set (Li et al., 2021). Verticillium dahliae (Vd), a soil-borne fungus with a wide range of hosts and high pathogenicity, is the primary cause of VW disease in cotton regions (Shaban et al., 2018). It is worth noting that the Vd primarily infects cotton plants from the root systems upward. Therefore, the detection of VW is of significant importance for reducing the devastation and economic loss.

The traditional method, such as the polymerase chain reaction procedure (Altaae, 2019), for detecting VW disease in cotton is the chemical detection method. These methods are destructive and relatively time-consuming. With the advancement of technology, some non-destructive detection techniques have been brought up, such as hyperspectral imaging and thermal imaging (Poblete et al., 2021; Yang et al., 2022). However, they can only detect VW based on symptoms in the above-ground parts of the plant. Since VW is infested from the roots, detection from the roots can be better for early detection.

Researchers have proposed various non-destructive methods to study the root systems, such as hydroponics, water-cooled gel culture, and computed tomography (CT) imaging. CT can detect roots *in situ* in soils such as wheat (Gregory et al., 2003), corn (Lontoc-Roy et al., 2006), and rice (Rogers et al., 2016). However, the similar absorption coefficients of soil and roots made it difficult for CT to distinguish them (Wu et al., 2018). In recent years, Magnetic Resonance Imaging (MRI) has been applied to the non-destructive inspection of plant roots. The principle of MRI is to obtain information by acquiring magnetic resonance signals at various locations within a magnetic field and then reconstructing the image of the object’s interior. The technique is extremely effective at detecting hydrogen atoms within a substance. During imaging, the signal intensity of spatial voxels is proportional to the number of hydrogen atoms present in the sample (Kumar Patel et al., 2015; Li et al., 2018; Lu et al., 2019). Both CT and MRI could produce tomographic images, but the results of experiments indicated that MRI provides greater root systems detail (Metzner et al., 2015). MRI has a high resolution, a variety of imaging parameters, the ability to choose any angle and dimension, and no radiation damage to the sample. Compare to medical MRI instruments, low-field nuclear magnetic resonance (LF- NMR) used in this paper is much cheaper. LF- NMR instrument has been widely used in agricultural science, including wheat(Chao et al., 2020), rice (Song et al., 2021), maize (Song et al., 2022), etc. In addition, the imaging parameters of medical MRI instruments are fixed parameters pre-set to obtain images of the inside of the body, while the parameters of LF-MRI can be flexibly adjusted. Therefore, LF-MRI technology is available for early, *in situ* detection of plant root diseases.

Since the morphology of plant roots changes after being affected by pathogens and external stresses, the morphological characteristics of roots in MRI images can be used to detect plant root diseases. Si mone Schmittgen et al. found by MRI that the volumetric growth of the taproot had already started to decrease on the fourteenth day after foliar Cercospora inoculation (Schmittgen et al., 2015). C.Hillnhütter et al. used MRI to non-invasively detect subsurface symptoms of sugar beet crown and root rot caused by sugar beet cyst nematodes and rhizobia. Lateral root development and sugar beet deformation were evident on MRI images of beet cyst nematode-infected plants 28 days after inoculation compared to uninfected plants (Hillnhuetter et al., 2012). Nowadays, some scholars have used deep learning and transfer learning to segment the plant root system and detect plant disease based on leaf image data. With a public dataset of 54,306 diseased and healthy plant leaves that were collected under controlled conditions, Sharada P.Mohanty et al. trained deep convolutional neural networks (CNN) and employed transfer learning to identify 14 crops and 26 diseases (or lack thereof) (Mohanty et al., 2016). Both (Wang et al., 2022) and (Guo et al., 2022) works of literature improved Swin Transformer (SwinT) to achieve the detection of plant diseases with an accuracy of 98.97% and 98.2%, respectively.

Compared with existing models, there were two difficulties in this paper. First, this paper studied the transverse section of the root system, which was different from the features of the longitudinal pictures of the root system in previous works. These segmentation models could not directly extract the features of root system cross-section in MRI images well. And the ratio of pixels occupied by the root system and soil studied in this paper was too disparate, which made the existing advanced segmentation models only segment the soil correctly and unable to capture the features of the root system. Second, existing disease detection models were mainly for RGB images of leaves and stems. However, in this paper, MRI images were grayscale images, which had less information than RGB images. Moreover, the disease features of leaves and stems were more numerous and obvious than those of roots. The MRI images of cotton roots could not provide so many features information on which the existing classification models were based. And the number of images of healthy and diseased samples is different, which can lead to a large loss in the model training process.

In this paper, the main purpose was to investigate the feasibility of MRI-based detection of VW infestation from cotton roots system. The specific objectives included the following: (1) denoise MRI images of cotton root to improve the signal-to-noise ratio of the images; (2) modify the MRI images segmentation model for obtaining the root target; (3) improve the image classification model to classify root MRI images between healthy and infected by Vd.

The main contributions include the following:

- The influence of pre-processing methods of cotton root MRI images was compared.
- We proposed the segmentation model and early disease detection model applicable to the MRI images of cotton roots. These models addressed the problems of unbalanced soil and root pixel scales and small data sets.
- Compared with other advanced models, our new models showed better robustness and extensibility. This demonstrated that early detection of cotton VW based on cotton root MRI images and deep learning was feasible.

The structure of the remaining portion of this paper is as follows: Section 2 describes the materials and methods. Section 3 explains the results and provides a discussion, and finally, conclusions are given in Section 4.

## 2 Materials and methods

### 2.1 Sample preparation

**Figure 1:**
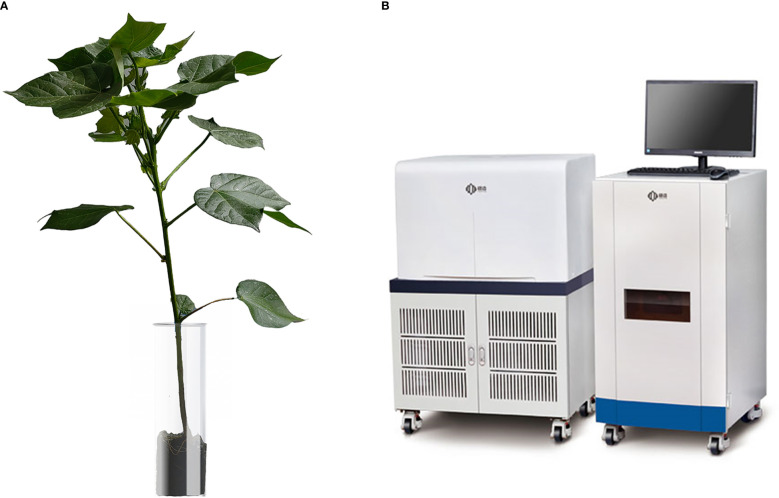
**(A)** Cotton experimental samples; **(B)** The low-field MRI instrument used in this paper.

In May 2022, the experiment was conducted at the college of Biosystems Engineering and Food Science at Zhejiang University in Hangzhou, Zhejiang province, China. The cultivar of cotton and oilseed rape were tested: Xinluzao 45 and Zhongshuang11, respectively. Cotton and oilseed rape seeds and the conidia solution of Vd were provided by the Agricultural College of Shihezi University, China. The concentration of conidia of Vd in the solution was 10^6^ conidia per ml. The cotton was divided into two groups: an experimental group and a control group of 20 plants each. These two groups received identical quantities of watering and fertilization. Each cotton plant in the experimental group was injected with 40 ml of a conidia solution. The control group was replaced with an equal amount of sterile water. After inoculation, the cotton was transferred to a greenhouse with daytime temperatures of 26°C and nighttime temperatures of 24°C and 60% humidity. MRI images of the root systems of 10 healthy and 10 infected cotton plants were collected on both day 15 and day 45 after inoculation. To avoid the effect of high soil moisture content on MRI imaging, the cotton was not watered for 48 hours before the formal MRI experiment. If the soil has high water content, it will be difficult to distinguish between the soil and tiny lateral roots. As shown in **Figure 1B**, a low-field magnetic resonance instrument (MesoMR23-060V-I, Niumag Co., Ltd., Suzhou, China) was utilized to acquire MRI images of cotton root systems. The low-field MRI device cannot collect images of targets smaller than 1 mm. The instrument relies primarily on the moisture signal for imaging. The more moisture a sample contains, the brighter it appears in the MRI images.

### 2.2 MRI images acquisition and data set division

Image acquisition: As shown in **Figure 1A** the entire cotton plant, including the soil, was placed in a 60 mm sample tube made of temperature-resistant quartz material. Spin-echo (SE) sequences were used to acquire axial MRI images. To obtain higher-quality MRI images, the following imaging parameters of the SE were optimized based on imaging quality and imaging time: TR (Repetition Time) = 1100 ms, TE (Echo Time) = 18.14 ms, Averages (Accumulation times at pre-scan) = 4, Slice thickness = 2 mm, Slice gap = 0.5 mm. The 2D Fourier transform reconstruction method built into the imaging software is used to reconstruct the image, after which 256×256 grayscale images were saved. 1191 MRI images were obtained, including 635 images of healthy roots and 556 images of infected roots. Samples were also collected from 2 healthy rape roots that had been growing for about 20 days, with a total of 32 MRI images.

Data set division: The dataset was divided based on the proportion 8:2 in this paper. In the image segmentation task, five healthy cotton root systems and five root systems infected with Vd were randomly selected. The 315 images of these ten cotton root systems were collected and utilized as a dataset for the segmentation task after being denoised. 252 images were used as the training set and 63 images were treated as the testing set. In the image classification task, 1191 images were used as the dataset, the training set consisted of 953 images and the testing set contained 238 images.

### 2.3 Data analysis

#### 2.3.1 Fine-tuning

Existing models in supervised learning require large quantities of labeled data, computational time, and resources. To save time and effort, transfer learning for deep learning is gaining more and more attention (Jiang et al., 2022). Transfer learning aims to apply knowledge or patterns acquired in one domain or task to a distinct but related domain or problem. Fine-tuning model is a method of transfer learning. The model parameters of pre-trained models are superior to those obtained by others after training with some classic models (VGG16/19, ResNet) and utilizing large datasets as training sets (ImageNet, COCO) (Hasan et al., 2022). In this experiment, both MRSwinUNet and MRResNet models utilized the fine-tuning method. After retaining the architecture of the model, the model was retrained using the initial weights of the pre-trained model to fine-tune.

#### 2.3.2 Loss function

The Focal loss (Lin et al., 2017) is a loss function that deals with the imbalance of sample classification. It focuses on adding weights to the losses corresponding to the samples according to the ease of sample discrimination, i.e., adding smaller weights to the samples that are easy to distinguish and larger weights to the samples that are difficult to differ. The Focal loss function was improved from the cross-entropy loss function. As in Equation (1)


(1) $$CE\left(p,y\right)=\{\begin{array}{c} -\log(p),\;\;\;\;\;\;if\;y=1\\ -\log\left(1-p\right),\;\;\;\;\;otherwise\; \end{array}$$

Here, ***y*** takes values of 1 and -1, representing the foreground and background, respectively. ***p*** takes values ranging from 0 to 1 and is the probability that the model predicts belonging to the foreground.

Next, as shown in Equation (2), a function on ***p*** is defined.


(2) $$p_{t}=\{\begin{array}{c} p,\;\;\;\;if\;y=1\\ 1-p,\;\;otherwise \end{array}$$

The combination of equation (1) and equation (2) leads to the simplified equation (3).


(3) $$CE\left(p,y\right)=CE\left(p_{t}\right)=-log\left(p_{t}\right)$$

To solve the positive and negative sample imbalance problem, a weighting factor **α** is introduced belonging to [0,1]. When it is a positive sample, the weighting factor is **α**, and when it is a negative sample, the weighting factor is 1-**α**. The loss function can be rewritten as:


(4) $$CE\left(p_{t}\right)=-\alpha_{t}log\left(p_{t}\right)$$

Formula (4) is called balanced cross entropy(BCE) loss and is the baseline for proposing Focal loss.

BCE loss does not distinguish between simple or difficult samples. When the number of easy-to-distinguish negative samples is super high, the whole training process will revolve around the easy-to-distinguish negative samples, which will in turn swamp the positive samples and cause large losses. Therefore, a modulation factor is introduced here to focus on the hard-to-score samples with the following formula (5).


(5) $$FL\left(p_{t}\right)=-{\left(1-p_{t}\right)}^{\gamma }log\left(p_{t}\right)$$

***γ*** is a parameter in the range [0, 5]. (1-***p_t_***) ***^γ^*** can reduce the loss contribution of the easy-to-score samples and increase the loss proportion of the hard-to-score samples. When ***p_t_*** tends to 1, which means that the sample is easily distinguishable. Then the modulating factor (1-***p_t_***) ***^γ^*** tends to 0, which means that it contributes less to the loss, i.e., it reduces the proportion of loss of the easily distinguishable sample. Small ***p_t_*** means that if a sample is divided into positive samples, but the probability that the sample is positive is particularly small, the modulating factor (1-***p_t_***) ***^γ^*** tends to 1, which does not have much effect on the Loss.

By balancing the above for positive and negative samples as well as difficult and easy samples, the final Focal loss formula (6) should be obtained.


(8) $$FL\left(p_{t}\right)=-\alpha_{t}{\left(1-p_{t}\right)}^{\gamma }log\left(p_{t}\right)$$

The imbalance in the number of positive and negative samples can be suppressed by ***α_t_***. And the imbalance in the number of simple or difficult-to-distinguish samples can be controlled by ***γ***. In this experiment, ***γ*** is 2 and ***α_t_*** is 0.25.

#### 2.3.3 Segmentation models

A hierarchical transformer called SwinT has been proposed (Liu et al., 2021), which was based on shift windows to implement the computation. The move operation allowed adjacent windows to be interacted with, significantly reducing the computational complexity. Compared with CNN, it showed competitive or even better performance on various visual benchmarks.

SwinUnet (Cao et al., 2021) was based on the SwinT network design for the image segmentation task, having transformer modules similar to the UNet structure. **Supplementary Figure 1A** represents the structure diagram of SwinT for the classification of the ImageNet dataset. And **Supplementary Figure 1C** depicts two SwinT modules connected in series, like a traditional multi-headed self-attentive (MSA) module structure’s construction on shifted windows. Each SwinT module includes a layer normalization layer (LN), a MSA module, a residual connection, and a multilayer perceptron with an activation function. In two consecutive transformer modules, a window-based multi-headed self-attentive (W-MSA) module and a shifted-window-based multi-headed self-attentive module (SW-MSA) are applied, respectively. ${\hat{z}}^{l}\;$ and *z^l^* denote the ML of the (SW-MSA) module and the 1 th block, respectively. The number of operations required to compute the correlation between two locations did not increase with distance, which made it possible to capture global semantic information more efficiently. In this paper, three major improvements were made to the SwinUNet model to obtain the model named MRSwinUNet.

First, We used transfer learning to better train the model. The specific step of the fine-tuning technique for transfer learning was to first preserve the original structure and then train with pre-trained weights. This improvement saved time on label annotation and reduced the requirement for the number of datasets. Next, the BCE loss function was replaced by the Focal loss function, which could distinguish the difficulty of segmented samples. A higher weight was given to the more difficult segmented roots, while a lower weight was given to the easily segmented soil pixels. The problem of large differences in the proportion of pixels occupied by cotton roots and background soil was solved. Finally, the AdamW optimizer was applied to improve the performance of the network.

#### 2.3.4 Classification models

The residual block structure of the ResNet network was proposed to solve the problem of gradient disappearance or gradient explosion. At the same time, it also addressed the issue of deeper levels leading to network performance degradation. Therefore, ResNet34 (He et al., 2016) network was chosen as the classification model based on the size of our dataset and the network performance of the devices used. **Supplementary Figure 2C** is a specific presentation of the residual block in **Supplementary Figure 2A**. In **Supplementary Figure 2C**, the feature matrix obtained after a series of convolutional layers on the mainline is summed with the input feature matrix, which is then output by the activation function. The output feature matrix shape of the main branch and shortcut must be the same. Formally, the desired underlying mapping is denoted as **H**(**x**) and the stacked nonlinear layers are made to fit another mapping: **F**(**x**) = **H**(**x**) - **x**. The original mapping is reshaped as **F**(**x**) + **x**. It is easier to optimize the residual mapping than to optimize the original, unreferenced mapping. In this work, we made three improvements based on the ResNet model using the cotton root MRI image dataset. The model named MRResNet was obtained afterwards.

We used transfer learning and changed the loss function of ResNet to Focal loss function, and replaced the original optimizer with AdaBound to solve the problem that the MRI images of cotton roots have less information than the RGB images of leaves or stems. The issue of different number of MRI images for healthy and diseased samples was also addressed.

The training parameters for the segmentation and classification network models are shown in **Table 1**.

**Table 1:** The segmentation and classification network model training parameters.

Items	Segmentation values		Classification values	
	original model	MRSwinUNet	original model	MRResNet
Learning rate	0.0001	0.0001	0.0001	0.0001
Optimizer	SGDM	AdamW	SGDM	AdaBound
Num_workers	4	4	4	4
Loss function	BCE loss	Focal loss	BCE loss	Focal loss
Epochs	100	100	10	10

### 2.4 Model evaluation and software

This paper evaluated the denoising model using the peak signal-to-noise ratio (PSNR) and structural similarity (SSIM) indices. Given a clean image I and a noisy image K of size m×n, the mean square error (MSE) and PSNR is defined as:


(4) $$MSE=\frac{1}{mn}\sum_{i=0}^{m-1}\sum_{j=0}^{n-1}{\left[I\left(i,j\right)-K\left(i,j\right)\right]}^{2}$$

(5) $$PSNR=10\times {log}_{10}\left(\frac{MAX_{I}^{2}}{MSE+\varepsilon }\right)$$

where $MAX_{I}^{2}$ is 255. ε is a very small constant that prevents the denominator from being zero. SSIM indicates the degree of similarity between two images. The definition is as:


(6) $$SSIM\left(x,y\right)=\frac{\left(2\mu_{x}\mu_{y}+c_{1}\right)\left(\sigma_{xy}+c_{2}\right)}{\left(\mu_{x}^{2}+\mu_{y}^{2}+c_{1})(\sigma_{x}^{2}+\sigma_{y}^{2}+c_{2}\right)}$$

where x and y are two signal indicators, *μ_x_* and *μ_y_* represent the means of x and y respectively, and *σ_x_* and *σ_y_* represent the standard deviations of x and y, respectively. *σ_xy_* represents the covariance of x and y. And *c*_1_,*c*_2_,*c*_3_ are constants to avoid systematic errors brought by a zero denominator.

In the segmentation task, the Dice coefficient (1), mean Intersection over Union (mIoU), Recall, and Precision metrics were used. And Accuracy, F1 score, Recall, and Precision metrics were used in the classification task.


(7) $$mIoU=\;\frac{1}{k+1}\sum_{i=0}^{k}\frac{TP}{TP+FP+FN}$$

(8) $$Recall=\;\frac{TP}{TP+FN}$$

(9) $$Precision=\;\frac{TP}{TP+FP}$$

(10) $$F1\;score=\;\frac{2\times Precision\times Recall}{Precision+Recall}$$

(10) $$Accuracy=\;\frac{TP+TN}{TP+FP+TN+FN}$$

***TP***, ***TN***, ***FP***, and ***FN*** indicate the number of true positives, true negatives, false positives, and false negatives, respectively. ***k*** is the total number of categories to be segmented.

The cotton root systems *in situ* images were annotated by the lasso tool of Adobe Photoshop CC2020 in the segmentation task. The segmentation and classification models were developed using the deep learning framework PyTorch (version 1.7.1). All models were generated with PyCharm (version 2019.2.3). A custom-built workstation with 48 GB of RAM and two GTX 1080 Ti graphics cards (NVIDIA, California, United States) was utilized.

## 3 Results and discussion

### 3.1 Denoising of the cotton root's MRI images

Image denoising could reduce the damage of noise to make the root system features clearer. In this paper, the parameters were optimized and the best parameter results were obtained for three models NLM, GLPF, and MF. **Table 2** details the comparison of the effects of each model.

**Table 2:** The denoising results of the MRI images of the cotton root systems.

Index	Optimal results	PSNR(dB)	SSIM
NLM	h = 10 Search windowsize = 21 x 21 Patch window size=3	32.776	0.952
GLPF	Kernel size = 5 x 5	24.643	0.882
MF	Kernel size =7 x 7	22.650	0.807

It is well known that a higher PSNR value represents a cleaner image. SSIM ranges from 0 to 1, with values closer to 1 indicating more image detail retention. NLM had the highest PSNR score and SSIM with 32.776 dB and 0.952, respectively. The PSNR score of GLPF and MF did not exceed 30 dB. Meanwhile, the SSIM index of GLPF and MF did not exceed 0.9.

**Figure 2:**
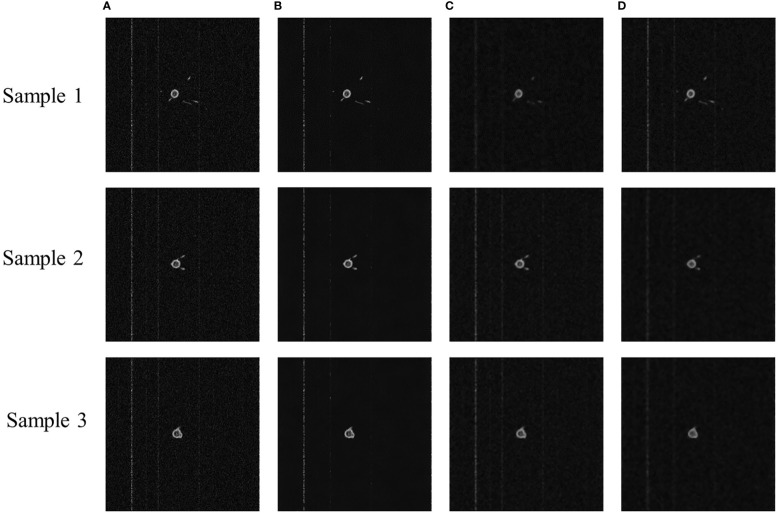
Sample images of denoised MRI images of cotton root system. **(A)** Original MRI image, **(B)** NLM denoised image, **(C)** GLPF denoised image, and **(D)** MF denoised image.

To examine the effect of denoising each model more visually, **Figure 2** presents the sample images for each model. NLM appeared the least noisy, with a clear background and more complete details. The denoising of GLPF and MF blurred the image and left the details incomplete.

Based on the PSNR and SSIM metrics and the subjective judgment of the vision, NLM had the best denoising effect and the most detail retention. It was because it had the ability to calculate the required pixels by weighted averaging of the entire pixels of the image, thus reducing the loss of image details. Since the noise was primarily concentrated in the high-frequency band, GLPF filtered the noise information to make the image smooth. But it also blurred the image. Additionally, MF also made the image more blurred. In this paper, it was considered that the image blurring and detail loss caused by GLPF and MF denoising processes were unacceptable. Therefore, the NLM model was chosen to denoise the MRI images.

### 3.2 Segmentation of the cotton root’s MRI images

In the segmentation task, the clean images after denoising were segmented to extract the root systems region, which was beneficial for the subsequent classification of the root systems. And the images were segmented at the pixel level.

**Table 3** outlines the segmentation results. The Dice coefficients and mIoU of both SwinUNet-B and TransUNet-B models were 0.5, and their precision and recall were both 0. It indicates that SwinUNet-B and TransUNet-B are not directly applicable to the segmentation task of cotton root MRI images. After improving the model, MRSwinUNet and TransUNet-D performed well with all metrics close to each other. However, the training time of MRSwinUNet was longer than the MRSwinUNet. This was a big drawback of the TransUNet model. So our MRSwinUNet model had the best overall performance. Compared with the original model SwinUNet-B, the Dice coefficient and mIoU of out model increased by 46% and 44%, respectively.

**Table 3:** The segmentation results of the MRI images of the cotton root systems.

model	Dice	mIoU	Precision	Recall	Time(s)
TransUNet - B	0.50	0.50	0	0	3360
TransUNet - D	0.96	0.93	0.95	0.90	3624
SwinUNet - B	0.50	0.50	0	0	1685
SwinUNet - D	0.95	0.90	0.89	0.90	1683
MRSwinUNet	0.96	0.94	0.91	0.95	1659
MRSwinUNet- Canola	0.93	0.90	0.95	0.89	-

The symbol "-" means no calculation time.

**Figure 3:**
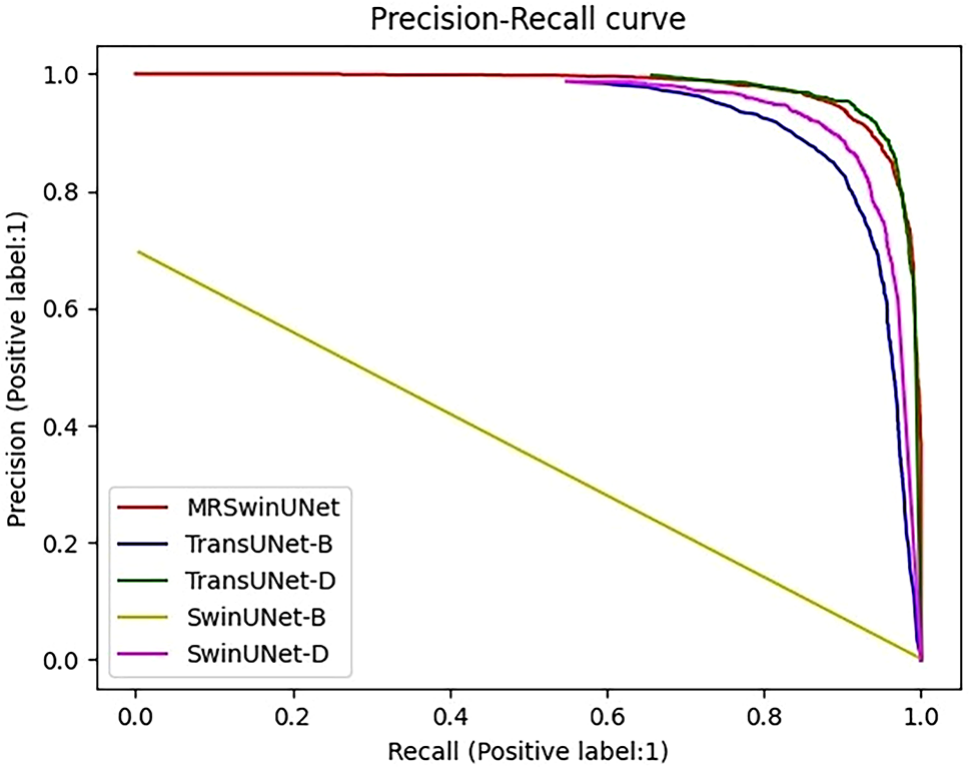
Precision-Recall curve. The label is 1, which means the infested root system is the positive sample. MRSwinUNet is our improved segmentation model. The TransUNet-B and TransUNet-D represent the TransUNet models using BCE loss and SGDM, Dice loss, and Adam, respectively. SwinUNet-B and SwinUNet-D represent the SwinUNet models using BCE loss and SGDM, Dice loss, and Adam, respectively.

To further demonstrate the results in **Table 3**, Precision-Recall (PR) curves were given in this paper. In the PR curve image, the closer the curve is to the coordinate (1,1), the better the performance. In **Figure 3**, TransUNet had the best PR curve, but MRSwinUNet’s PR curve was right next to it and got just as good results. And the original model SwinUNet performed the worst.

According to the observed experimental images, it was known that the ratio of pixels occupied by the root system and the soil was approximated at a minimum of 1:16383. However, the original SwinUNet-B and TransUNet-B models were trained by assigning the same weights to the root system and the soil. In this case, the original loss function and the optimizer only guided the model to correctly segment the soil pixels and could not work for the root system roots. In addition, although the metrics of both TransUNet-D and SwinUNet-D were improved, they were still not as good as the combined performance of MRSwinUNet. It was probably due to the reason that the Dice loss function and Adam optimizer did not perform as well as the Focal loss function and AdamW used in this paper.

**Figure 4:**
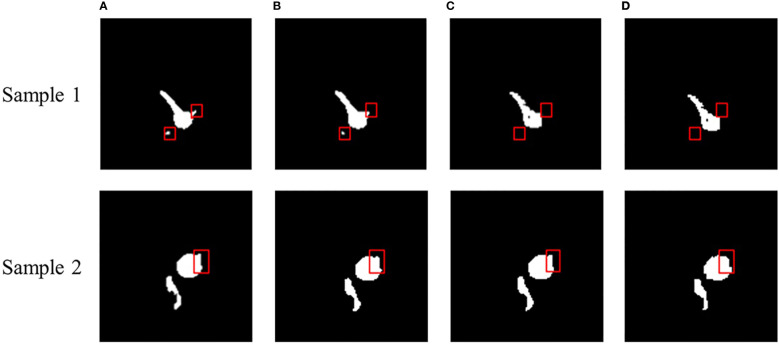
Sample segmentation effect of MRI images. **(A)** Real label, **(B)** MRSwinUNet, **(C)** TransUNet-D, **(D)** SwinUNet-D.

To better demonstrate the segmentation effect, we performed a visual evaluation. **Figure 4** presents the representative figure of root segmentation effect of SwinUNet-D, TransUNet-D, and MRSwinUNet. The findings demonstrate that all three sample maps differed somewhat from the accurate label maps in detail. For instance, inside the red box of **Figure 4**, the SwinUNet-D barely segmented any effective information on the root. The resultant map of MRSwinUNet segmentation was similar to the original label with sufficient detail. So MRSwinUNet was considered to be the most optimal model for the overall performance of the segmentation task.

To investigate the scalability of the MRSwinUNet model, we selected 32 MRI images of canola obtained with the same acquisition method and preprocessing method. The trained MRSwinUNet model was used to segment the rape dataset, and the results were displayed in the MRSwinUNet-Canola model in **Table 3**, the Dice, mIoU, of the canola segmentation results were 0.93 and 0.90, respectively. In addition, its Precision score was 4% higher than that of the cotton dataset. This indicates that MRSwinUNet has better robustness and extensibility.

Previous research scholars (Shen et al., 2020; Kang et al., 2021; Lu et al., 2022; Zhao et al., 2022) have also done comprehensive studies on plant root segmentation. In this paper (Kang et al., 2021), the cotton mature root systems were used as the research object. They designed a semantic segmentation model of cotton roots *in-situ* images based on the attention mechanism. The precision and recall values were 8.7% and 4.8% higher than those in this paper, respectively. This would be due to the high resolution (10200×14039 dpi) of the root images they acquired, which was easy to identify and segment. In addition, they trained the model directly using their dataset. Although the training process took a lot of time, it facilitated the extraction of root features in the images and reduced segmentation errors.

### 3.3 Classification of the cotton root’s MRI images

**Figure 5:**
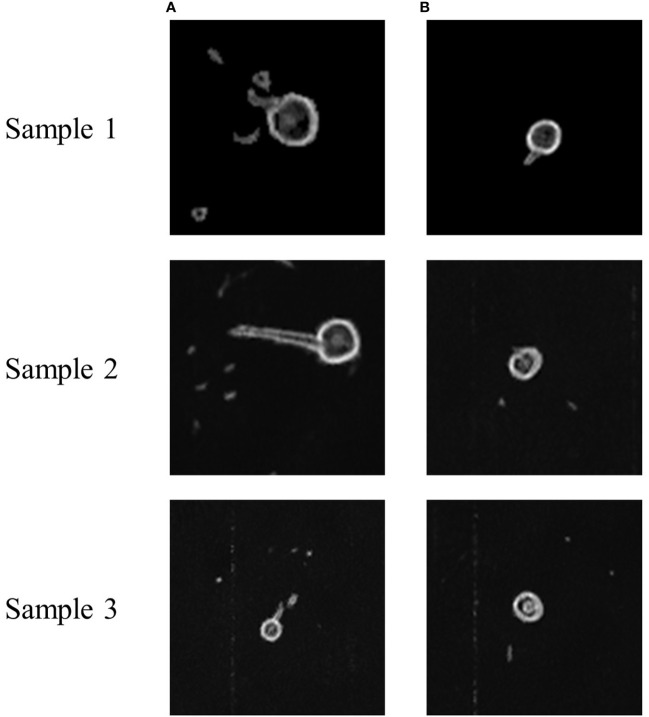
Sample MRI images of healthy and diseased roots of cotton. **(A)** Healthy cotton root system, **(B)** Infected cotton root systems. These sample images are from the same location of different root systems.

**Figure 6:**
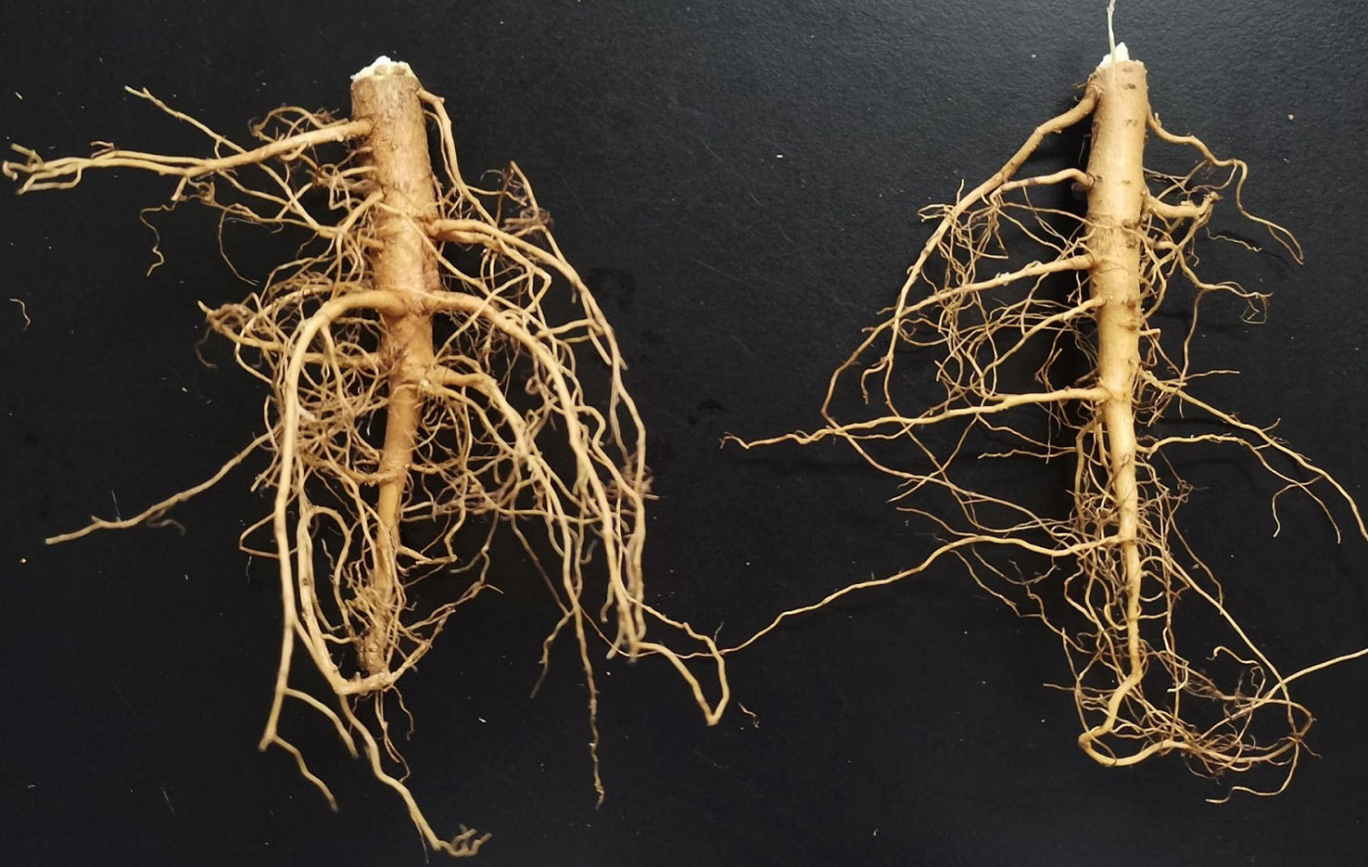
Samples of the cotton root system. The root on the left is healthy. The root on the right is affected by the Vd.

**Figures 5**, **6** show the MRI images of the healthy and infected root systems. The smallest root diameter that can be detected by the low-field MRI instrument used is 1 mm. This means that all the roots in the figure have a cross-sectional diameter greater than or equal to 1 mm. It should be noted that the healthy root systems had more branching cross-sections than the diseased root systems, which could be ascribed to the fungus also colonizing the ducts and secreting toxins that damage the cells (Bai et al., 2022; Lv et al., 2022; Ren et al., 2022; Sayari et al., 2022). Consequently, cell growth would be hindered, and the number of lateral roots reduces, which provides the possibility of classifying the MRI images of healthy and unhealthy root systems.

In **Table 4**, the results of all metrics of the original models (SwinT, Vgg16Net, ResNet) were unsatisfactory. It indicates that the original models cannot perform the classification task regarding the root MR images. Compared to the original model, the results obtained by our MRResNet using all five preprocessing methods were significantly improved. The highest accuracy was achieved when MRResNet used the dataset processed by denoising first and then segmenting, with 34.9% improvement over the original ResNet model, and 59% and 42% improvement for Recall and F1, respectively. When MRResNet was trained on the dataset processed in the other four ways, the results were all improved over the original model. But it was still lower than the results of the dataset processed by the denoising-only method, denoising first and then the segmentation method.

**Table 4:** Classification results of the MRI images of healthy versus Vd-infested cotton root systems.

Model	No.	Accuracy (%)	Precision	Recall	F1	Time(s)
SwinT	1	53.40	0	0	0	327
	2	53.40	0	0	0	331
	3	53.40	0	0	0	318
	4	53.40	0	0	0	327
	5	53.40	0	0	0	316
Vgg16Net	1	46.60	1	1	1	357
	2	53.40	0	0	0	359
	3	53.40	0	0	0	351
	4	46.60	1	1	1	352
	5	53.40	0	0	0	355
ResNet34	1	53.60	1	0.05	0.09	246
	2	58.00	1	0.56	0.74	270
	3	53.40	0	0	0	256
	4	60.10	1	0.41	0.58	268
	5	53.40	0	0	0	255
**MRResNet**	1	89.90	1	1	1	259
	2	**92.00**	1	0.96	0.97	267
	3	76.50	1	0.91	0.95	252
	4	**95.00**	**1**	**1**	**1**	268
	5	70.20	1	1	1	261

The column No. represents the datasets with different preprocessing methods. Five numbers from 1 to 5 represent the original dataset, denoised dataset only, segmented dataset only, denoised-resegmented dataset, and segmented-redenoised dataset, respectively.

**Figure 7:**
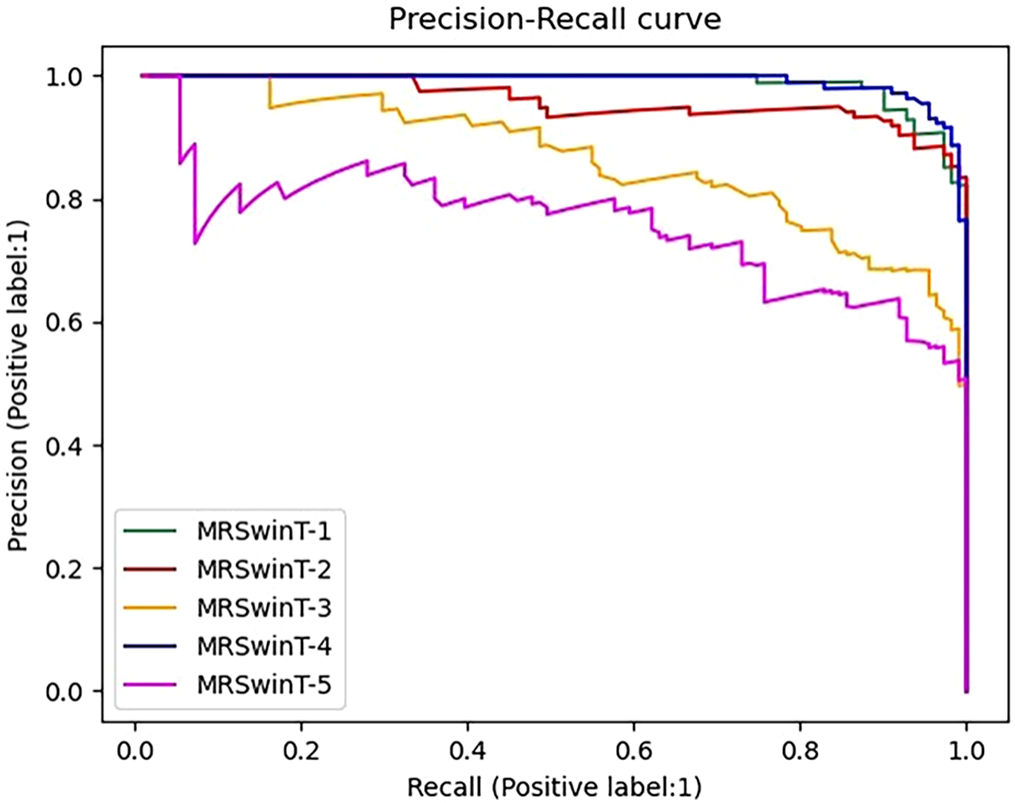
Precision-Recall curve. The label is 1, which means the infested root system is the positive sample. MRSWinT-1, MRSWinT-2, MRSWinT-3, MRSWinT-4, and MRSWinT-5 represent the original dataset, denoised dataset only, segmented dataset only, denoised-then-segmented dataset, and segmented-then-denoised dataset, respectively.

To compare more comprehensively the effect of image preprocessing methods on the classification results of MRResNet models, PR curves were plotted. In **Figure 7**, the curve of the denoised and then-segmented dataset was closest to the coordinate (1, 1). This indicates that this dataset performs best in the classification task. From the results, it can be concluded that the denoised and then-segmented dataset worked best in classification model training. Because it filtered out the noise, reduced image pollution, and avoided the problem of blurred root features. Furthermore, the root targets were extracted precisely by segmentation, which made the root features more clearly. The denoised dataset performed the second best. This was because the image denoising process mainly filtered out the noise in the image, but some root features had weak signals that were not further extracted by segmentation, which caused the classification model to ignore this part of the signal. The bad thing was that the dataset with only segmentation and segmentation followed by denoising process methods lost the original root system features. The reason was that without noising processing, which made the image contaminated with noise, the segmentation model did not recognize the segmented features and lost the smaller but more important information of the signal, such as the lateral root cross-section.

In conclusion, MRResNet was considered the optimal model considering all model metrics, training time, and PR curves. The best way to process the dataset was to denoise the images first and then segment them.

Compared with the high accuracy of the existing literature (Li et al., 2020; Liang, 2021; Santos-Rufo and Rodriguez-Jurado, 2021; Sivakumar et al., 2021; Elaraby et al., 2022; Memon et al., 2022), the accuracy of the identification of roots suffering from cotton VW disease was about 4% lower in this paper. Studies in the literature have targeted leaves and stem with obvious disease symptoms, such as leaf yellowing and wilting. Thus, the accuracy was higher when it came to disease detection. However, this paper studied cotton root systems in the early stages of VW. Since the morphology of each cotton plant varied, the classification model probably misclassified healthy cotton with a small root system as diseased cotton or, conversely, misclassified diseased cotton with a well-developed root system as healthy cotton. These misclassifications resulted in a lower accuracy rate in this paper than in other literature. Nevertheless, the method in this paper still provided a new idea for the detection of cotton VW disease. After the root system was infested, it had already changed before the leaves turned yellow and wilted. In this situation, theoretically, the technique adopted in this paper could detect the disease much earlier.

### 3.4 Limitations and prospects

Image denoising and segmentation contributed to clean root systems MRI images, and deep transfer learning improved the ability to learn image features. The combination of these two approaches realized effective classify healthy and Vd-infested cotton roots. However, after being inoculated, the immune system of cotton was damaged. Along with that, there was a great possibility of infestation by other pathogens, which could be time-consuming and costly to identify. Considering the observation that cotton predominantly presented symptoms of VW when it developed, cotton VW was examined as the main disease in this paper. Besides, the number of lateral roots in this paper was only observed in 2D images. The changes in root morphology after infestation by Vd were not presented in full. In the future, we will continue to study the changes in the three-dimensional morphology of cotton roots after being infested with Vd. Finally, due to the lack of images of other plant roots affected by VW disease, there was no way to do experiments to further explore its robustness and scalability. In the future, we will collect more image data on plant roots suffering from VW disease, and thus build a robust and extensible model for the detection of VW disease.

## 4 Conclusions

In this paper, we first used cotton root cross-section LF-MRI images as samples to explore the feasibility of early nondestructive detection of VW disease in cotton using deep. First, the performance of three denoising models NLM, GLPF, and MF was compared, and the results showed that NLM had the best denoising effect. After that, the SwinUNet model was modified in three parts and obtained the MRSwinUNet applicable to the MRI image segmentation of the cotton root system. The Dice and mIoU of MRSwinUNet increased by 46% and 44%, respectively, over the original SwinUNet’s results. And it addressed the problem of unbalanced soil and root pixel proportions and reduced the effort as in the original model. MRSwinUNet also had a good segmentation effect on MRI images of the canola root system. Subsequently, NLM and MRSwinUNet were selected to denoise and segment the cotton root dataset respectively, and the classification datasets with five pre-processing methods were obtained. And then the original classification models (SwinT, Vgg16Net, ResNet) were chosen to classify cotton root images, but the results were extremely poor. Therefore, in this paper, we made improvements to the ResNet model to obtain the MRResNet model for cotton root MRI image classification. The results of five datasets were compared on the classification model, and showed that the first denoising and then segmentation treatment worked best. When MRResNet used the best dataset, its accuracy improved by 34.9% over the original model. Meanwhile, the recall and F1 improved by 59% and 42%, respectively. This demonstrates the feasibility of detecting cotton VW disease at an early stage using deep learning and MRI images of the cotton root system. The paper provides a new research idea for the detection of VW disease in cotton.

## Data availability statement

The raw data supporting the conclusions of this article will be made available by the authors, without undue reservation.

## Author contributions

WT, QX, and SC designed the study, conducted the experiment, and wrote the manuscript. LF and NW supervised experiments at all stages and performed revisions of the manuscript. PG and YH performed revisions of the manuscript. All authors contributed to the article and approved the submitted version.
