# Supplementary figures and images for: Early detection of cotton verticillium wilt based on root magnetic resonance images
Source: Front Plant Sci. 2023 Mar 20;14:1135718. doi: 10.3389/fpls.2023.1135718 (PMC10067745; doi:10.3389/fpls.2023.1135718)

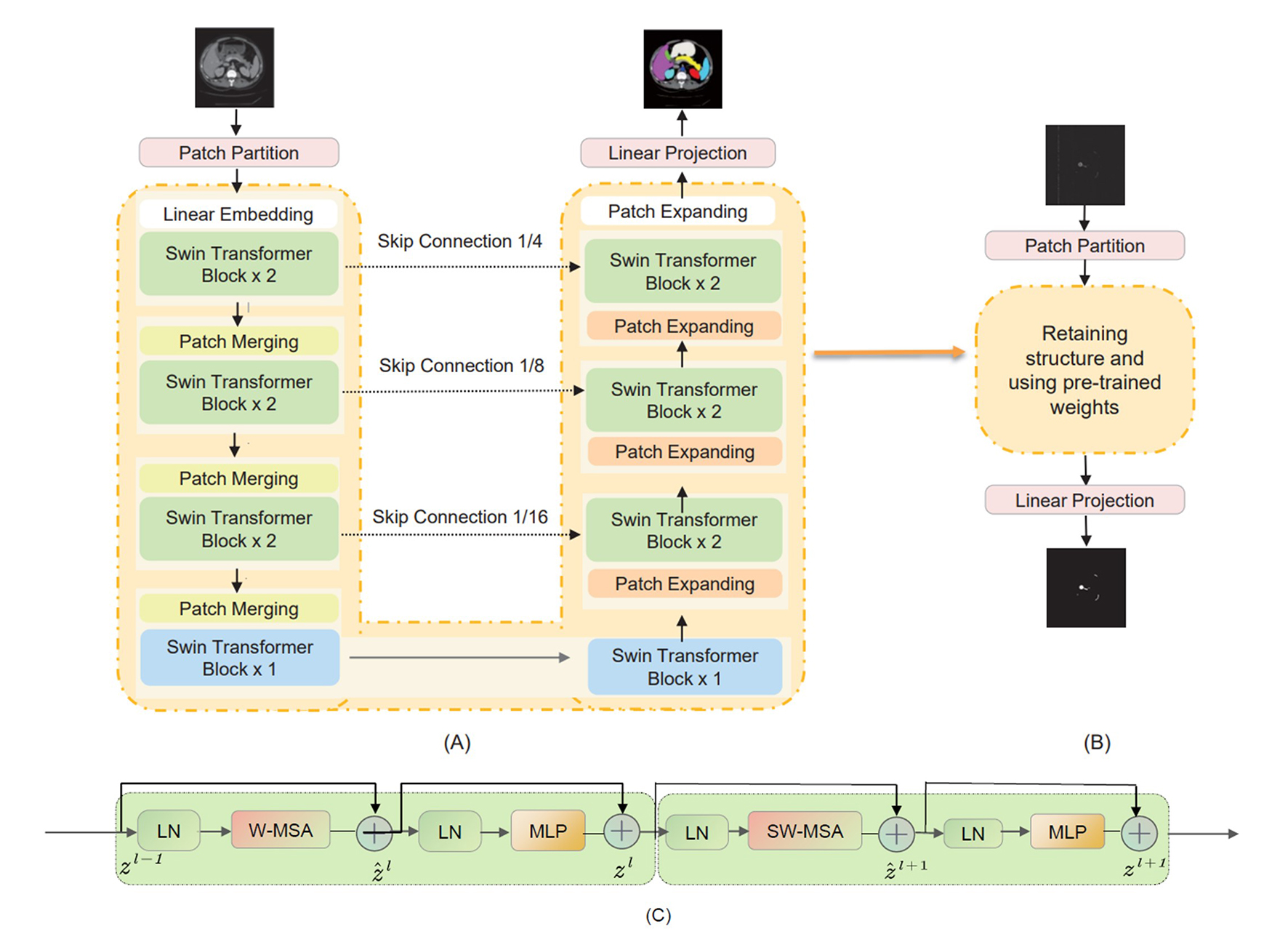

Supplement: Supplementary file 1 [file Image_1.jpg]

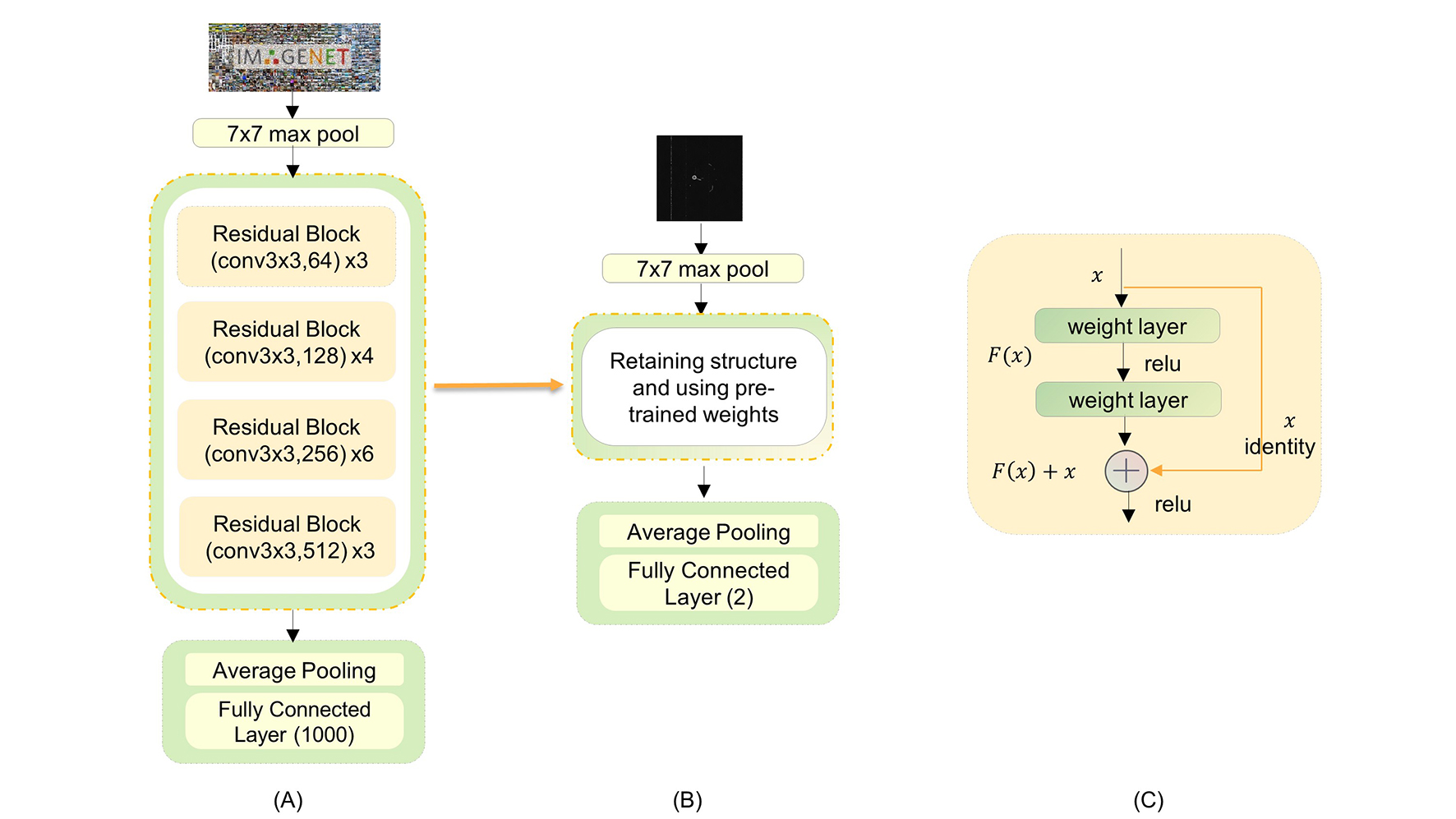

Supplement: Supplementary file 2 [file Image_2.jpg]
